# A Large-Scale Screening Responding Sporadic Epidemic of COVID-19 in China by an Integrated Health-Care System

**DOI:** 10.5334/ijic.5941

**Published:** 2022-03-23

**Authors:** Xuru He, Xizhuo Sun, Fangfang Gong, Wenhai Li, Jinchun Lin, Hanqun Lin, Guangyu Hu

**Affiliations:** 1 Department of Hospital Group Office, Shenzhen Luohu Hospital Group Luohu People’s Hospital (The Third Affiliated Hospital of Shenzhen University), Shenzhen, 518001, CN; 2 Community Health Management Center, Shenzhen Luohu Hospital Group, Shenzhen, 518001, CN; 3 Branch of Luohu District Hospital of Traditional Chinese Medicine, Shenzhen Luohu Hospital Group, Shenzhen, 518001, CN; 4 Institute of Medical Information/Center for Health Policy and Management, Chinese Academy of Medical Sciences and Peking Union Medical College, Beijing, 100730, CN

**Keywords:** COVID-19, large-scale screening, sporadic epidemic, integrated health-care system

## Abstract

**Introduction:**

In the post-pandemic period of COVID-19, the majority of cities in China try to balance the normalization of epidemic prevention and social-economic development. However, the appearance of asymptomatic infected patients poses threats to public health, which might be infectious without clinical symptoms and only be detected by testing approaches.

**Methods:**

Along with the appearance of one symptomatic case, a regional large-scale screening program was carried out in Shenzhen City charged by a regionally integrated healthcare system. After describing the screening program, a retrospective cross-sectional study for the screening outcome and efficacy was conducted.

**Discussion:**

According to the screening results, the asymptomatic case was infectious and their close contacts should be quarantined cautiously as the close contacts of symptomatic cases. Besides, after integrating medical resources in Luohu district of Shenzhen, the medical capability of Luohu district improved greatly which could be demonstrated in inspection and organization abilities in this screening program.

**Conclusion:**

The large-scale screening contributed to preventing epidemic transmission. In the post-pandemic period, regular surveillance of asymptomatic cases and rapid response capability for emergent screening program are both crucial for the prevention and control of COVID-19 epidemic. The integrated healthcare system coordinating regional medical institutions and optimizing regional medical recourse has advantages to address public health emergencies.

## Introduction

### Background

The situation of imbalanced medical and health resources allocation has obstructed residents to receive proper medical treatment in China. On one hand, the financial rationing strategy supported by the government is affected by profit-oriented market economy, resulting in an imbalanced distribution in governmental subsidies among urban areas and rural areas, among large-scale public hospitals and regional hospitals with smaller sizes, and among public hospitals and primary care systems [1]. Previously, 80% of financial support from the government was for urban medical institutions, of which 80% of them was invested for large-scale public hospitals which have less accessibility for disadvantaged groups [2]. On the other hand, the medical human resources prefer to work for large-scale hospitals, especially provincial and municipal public hospitals, due to the differential distribution in financial rationing strategy and medical resources based on service volume and hospital level [3]. The hospital-centric health delivery system exacerbates the gaps of medical and human resources allocation between urban and rural areas, between public hospitals and primary care systems, and among hospitals with different sizes, which also exacerbates the gap of medical capacity for health sectors and lowers medical accessibility for residents [4]. It is one of the challenges to medical reform in China to enhance resources utilization and sharing mechanism among health sectors to increase the medical capacity, optimize working efficiency, and lower medical expenditure [3].

To counter the rising burden of a hospital-centric and treatment-dominated delivery system in China, the World Health Organization and World Bank cooperating with the Chinese government jointly published a report in 2016, which proposed to enhance China’s health-care system through a person-centered integrated care model [5]. As WHO defined, the integrated care system helps broader health and social care systems effectively respond to the diverse and complex health needs of people [6], to provide a continuum of care ranging from health promotion and preventive care to treatment services. As designed, it is essential for China to build a collaborative relationship between primary healthcare systems and hospital sectors to develop an integrated health system [3], so that health providers within integrated care system with the same goal can be organized to manage population health in a coordinate way rather than having strong incentives to compete with each other to maximize their profits in each health provider [5,7]. Therefore, vertical and horizontal integration can be realized in an integrated care system, which provides a chance to optimally allocate regional medical resources and human resources.

For example, a citizen integrated care system comprises regional public hospitals and community healthcare centers. The primary healthcare system, such as community healthcare stations or centers in urban areas, should be a foundation where health providers are closely connected and coordinated to offer continuing health management and health promotion for residents; while the hospitals should be more specialized in specific diseases treatment (such as acute and critical diseases, rare diseases, etc.) instead of normal discomforts or chronic disease. Thus, a mature integrated care system with has strong primary care system and sufficient utilization of medical resources could greatly strengthen the regional medical capability and working efficiency.

Since 2019, the Corona Virus Disease 2019 (COVID-19) epidemic has affected more than 210 countries [8] and caused more than 5 million deaths around the world, which is the most serious public health event in recent years [9]. Currently, the mainland of China has effectively controlled the outbreak of COVID-19, and the economic and social activities are gradually recovering; however, there are still risks of imported cases and sporadic local cases [10]. As the personal protective equipment (PPE) has gradually been neglected and the international flights have recovered during the resumption period of social and economic activities in the mainland of China, the clustered and sporadic epidemic situation has occurred continuously in which the integrated care system plays an important role in epidemic prevention and control. There were domestic confirmed cases in Yunnan, Harbin, Jilin, Beijing, Urumqi, and Dalian since April 2020, which disrupted the peace of returning to normal life and could further affect public health at the domestic level if the epidemic prevention and control measures are not taken at the first time. Also, the appearance of asymptomatic cases poses another threat to public health. On one hand, asymptomatic patients could only be confirmed by positive nucleic acid testing results without presenting clinical symptoms, which means that the diagnosis and quarantine of them might be delayed and thus provide virus a change to transmission [11]. On the other hand, truly asymptomatic patients and pre-symptomatic patients are difficult to distinguish since both of them are non-symptomatic. The pre-symptomatic patients among asymptomatic infection account for 48.8% [12] who have a higher viral nucleic acid content two days before onset [13], which means nearly half of non-symptomatic infections might develop symptoms later and become infection sources.

Since the epidemic of COVID-19 has a severe impact on global health and economic development, it is necessary to adopt useful measures to mitigate the spread in sporadic situations. Mass screening has been adopted as a preventive approach in post-pandemic to implement regularly for high-risk populations and as a controlling approach in a sporadic epidemic situation to screen out the infectious population in communities [14,15,16]. However, a large-scale screening program conducted in a short time is a great challenge to single medical institutions with limited local medical affordability, limited information sharing ability, and limited material reserve. It also requires completing personnel regulation, setting up temporary collection teams, carry out sampling and testing work in that short time. Containment of future outbreaks will critically depend on early testing in work sectors and geographical regions [15]. A single medical institution could not cope with a sporadic pandemic situation and successfully conduct a mass screening, while an integrated healthcare system has potential advantages in response to community outbreaks of highly infectious diseases (such as COVID-19) and their transmission.

This study shares an experience from an integrated healthcare system about the management of sporadic COVID-19 cases by the large-scale screening in social and economic recovery settings.

### Problem statement

Shenzhen, one of special economic zones in China, is adjacent to Hong Kong. In the post-pandemic period, numerous cross-border personnel commute between Shenzhen and Hong Kong, such as cross-border drivers who are responsible for the daily transportation of food and living materials. This group of people forces medical institutions in Shenzhen to formulate a management strategy for the sporadic epidemic situation.

On August 14, 2020, a salesperson in a supermarket in Luohu District of Shenzhen City was diagnosed with COVID-19. As the large visitor flowrate of the supermarket and the uncertainty of the infection source and time of the index case, an emergent large-scale screening program needed to be applied in the high-risk population. The Shenzhen government and relevant medical institutions immediately initiated prevention and control measures, and an integrated health-care system in Luohu District was mainly responsible for this screening program.

## Methods

It was designed as a retrospective and cross-sectional study for a regional large-scale screening for sporadic COVID-19 epidemic leaded by an integrated health-care system in Shenzhen, China.

**Figure 1:**
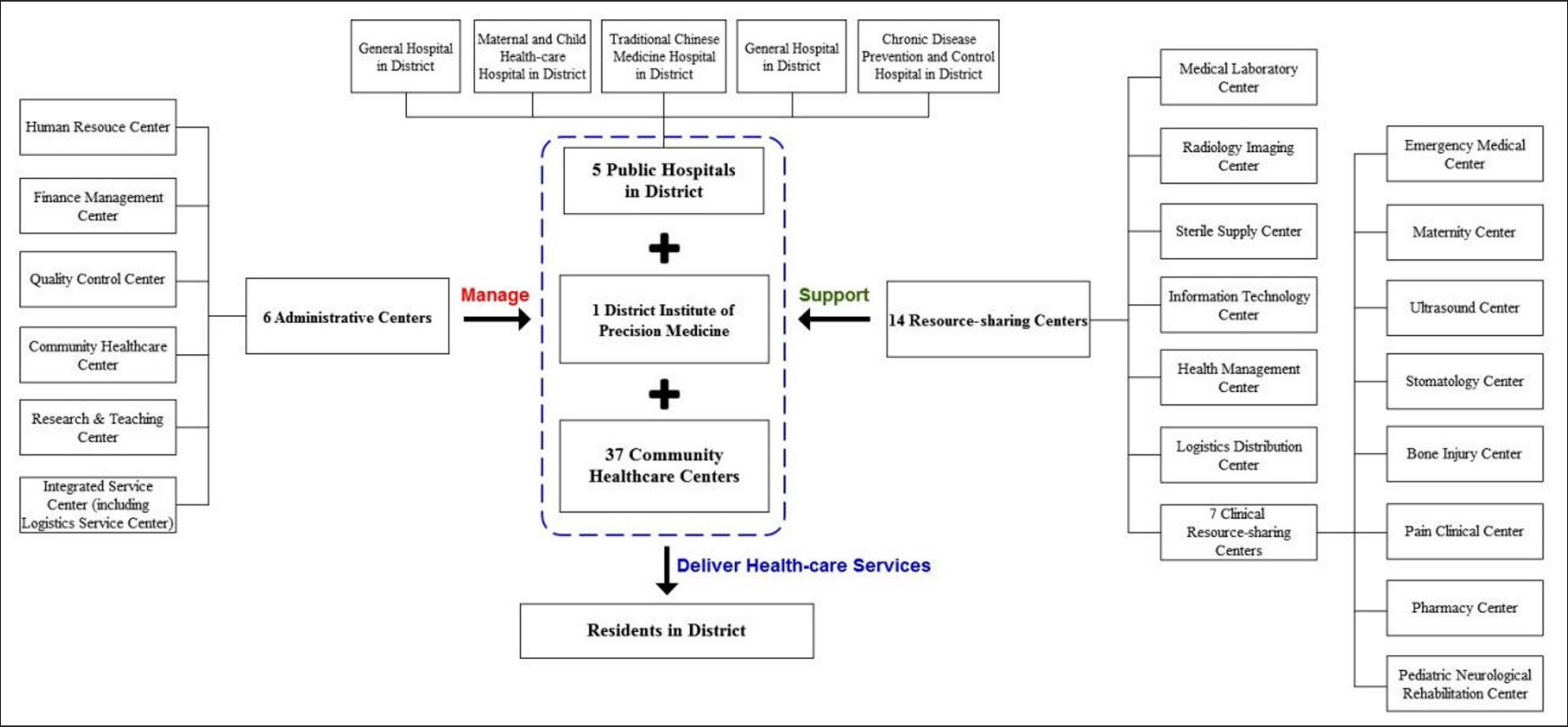
Construction of the integrated health-care system in Luohu District, Shenzhen City.

The integrated health-care system in Luohu District of Shenzhen City, the Shenzhen Luohu Hospital Group (after this referred to as the Hospital Group), which consists of 5 public hospitals and 37 community healthcare centers, and an institute of precision medicine in Luohu District (see ***Figure 1***). A person-centered integrated care model established in this system [5] aims to provide continuum care for residents with the range from health promotion and preventive care to treatment services throughout their whole life [17]. To deepen the integration among regional medical institutions, there are 14 resource-sharing centers and 6 administrative centers established in the Hospital Group after integrating regional personnel and materials resources from all medical institutions mentioned above. With this integrated health-care system, Luohu district can respond to public health emergencies effectively.

### Screening objectives

This screening program multicentred on the living and working places of the index case and the suppliers of the supermarket, covering 127 residential communities (or residential enclosures) as well as nearby office buildings, merchants, and construction sites. The screening program recruited residents living in the 127 residential communities, employees working in the nearby buildings, customers of the supermarket, and residents who had visited these areas within 14 days. Also, the environmental samples in screening areas were collected by medical staff for inspection.

### Diversity of screening strategies

A large-scale screening requires not only the sampling work to cover all the population in the required screening area, but also the collection and inspection of a large number of samples to be completed in a short time. Considering that the COVID-19 has an incubation period and the accurate infected time of the first domestic case remains unknown, it would appear missing infected case only based on once-time testing result. The previous studies also suggested that early quarantine conducted alone or combined with other interventions would be the most effective measure among contact tracing, screening, quarantine, and isolation [18]. Thereby, the Hospital Group designed a diverse screening strategy comprising different interventions to ensure coverage and accuracy according to the screening population or objective (see ***Table 1***).

**Table 1:** The screening strategies in large-scale screening program.


STRATEGIES	SCREENING OBJECTIVES	SAMPLING APPROACH	TESTING APPROACH	QUARANTINE REQUIREMENT

High-risk population	Close contacts of the index case, such as cohabiting family member, colleague, etc.	Single sampling	Daily nucleic acid test and once serological antibody test	14 days of centralized isolation in designated hotels

Sub-high-risk population	The cohabiting family members of the index case’s close contacts and neighbor in living or working place of the index case	Single sampling	At least 2 times of nucleic acid test and once serological antibody test	7 days in centralized medical observation points + 7 days at home

Community screening population	Residents in screening area, customers have been to the supermarket, etc.	Pooled sampling	Nucleic Acid Test	0

Accessible service	Inconvenient people, such as the elderly and the disabled	Depends	Nucleic Acid Test	0

Environmental sampling	External environment and product samples within screening areas	Single sampling	Nucleic Acid Test	–


Both the residents and environmental objectives were identified as screening targets since the virus has shown environmental resistance and could transmit through surfaces, water, air, and food [19]. Herein, the environmental samples were collected to undergo the nucleic acid test in the area of the living place, the workplace, and the suppliers of the supermarket. The screening population was divided into the high-risk population, sub high-risk population, community screening population, and inconvenient population. Screening measures were tactically increased in populations with a higher risk of exposure.

The high-risk population, including the close contacts of index diagnosed case, was required to isolate in designated hotels immediately for medical observation, and then underwent daily nucleic acid test via nasopharyngeal swab and one serological antibody test in the isolation duration.

The sub-high-risk population was defined as the cohabiting family members of the index case’s close contacts and neighbors in living or working place of the index case. The quarantine requirement was at least 7 days for centralized medical observation in designated hotels and 7 days for self-quarantine at home. Two times of nucleic acid testing and one serological antibody testing were necessary for them to be cleared.

As for the community screening population, who accounted majority, the sample collection sites were set up in residential communities for lower-risk residents to complete the nucleic acid testing without quarantine or isolation requirement. In addition to the traditional sampling sites, the mobile sampling teams were adopted for the inconvenient population. The Hospital Group arranged 2 mobile sampling vehicles to provide sample collection for the scattered residential buildings without functioning sample collection sites, which helped to increase the coverage of sampling population. It also could benefit from relieving the working pressure of traditional sampling sites. Meanwhile, the other mobile sampling teams undertook door-to-door nucleic acid sampling for the elderly and the disabled.

Notably, in terms of the sampling approach, because of the large number of samples, the single sampling and pooled sampling were conducted in parallel, which greatly speeded up the sample detection efficiency. The single sampling was used for high-risk and sub-high-risk groups, and 5 in 1 pooled sampling was used for community screening.

### The coordination in the integrated health-care system

To ensure the development and coordination of hospitals and community healthcare centers in the integrated care system, the resource-sharing centers, and administrative centers have been established since 2015, which were involved and contributed greatly in the whole screening procedure that helpfully increased the screening efficiency. The administrative centers are responsible for managing congener resources and allocating them to achieve maximum working efficacy. The resource-sharing centers focus on homogenizing the medical service and developing working capability within the Hospital Group by gathering congener resources. For example, the Human Resources Center (HRC) is established as an administrative center to manage the human resources in the integrated care system, which means the center can organize regional medical labors as a whole, allocated reasonably and managed scientifically. Similarly, the Logistics Services Center (LSC), as an administrative center, gathers congener resources to optimally allocate as HRC does. The Logistics Distribution Center (LDC), the Medical Laboratory Center (MLC), and the Information Technology Center (ITC) are resource-sharing centers that gather congener resources to maximize the working efficiency in resource distribution, inspection works, and IT services respectively, and also to guarantee the homogenization of medical and health service among all the health sectors in Hospital Group.

**Figure 2:**
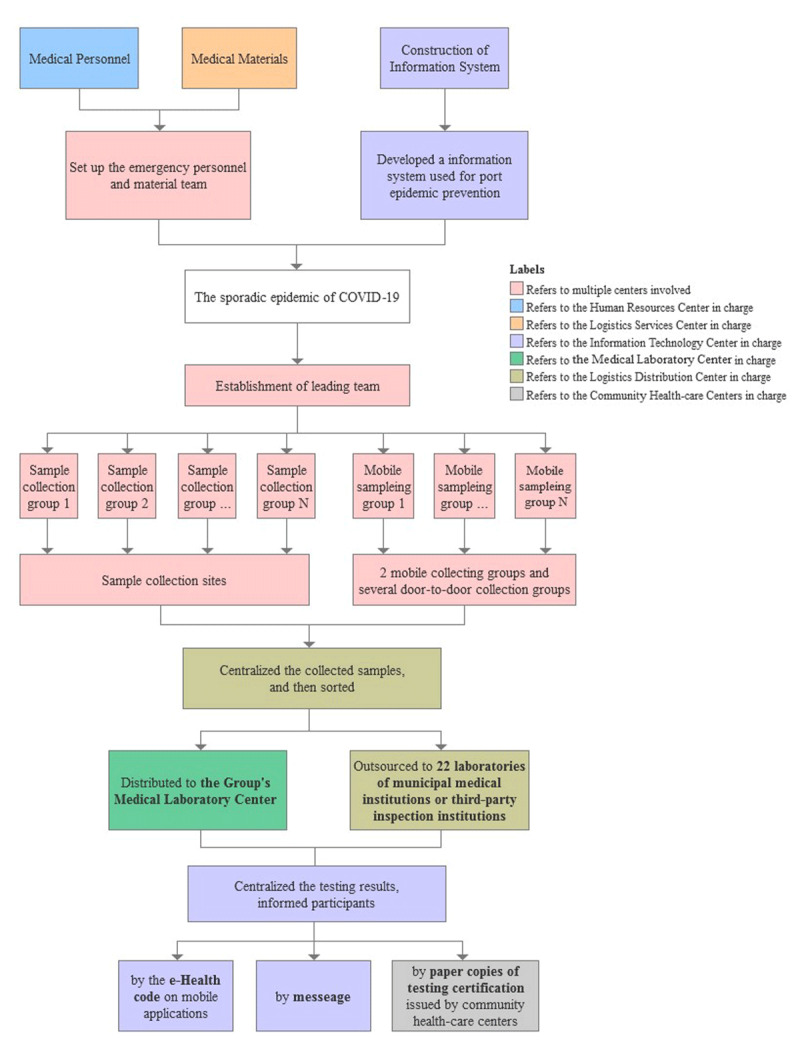
The flow chart of large-scale screening program conducted by the Hospital Group.

With the administrative centers and resource-sharing centers, large-scale screening can process successfully. The large-scale screening program had three main stages (see ***Figure 2***) and the details of each stage are shown in Supplementary Appendix 1. In the preparedness stage, HRC and LSC integrated regional medical personal and material resources, and set up the name list of emergency response team with a clear division to make sure sufficient resources to cope with emergent pandemic outbreak. Besides, the informational system used in normal port epidemic prevention supported the emergent screening program for the sporadic epidemic.

Once the appearance of sporadic cases, the Hospital Group and the local government established a leading team to direct the screening program. The emergency response teams reacted immediately and divided into sample collection groups for collecting nasopharyngeal swabs and blood samples for testing. Before distributing to inspection, LDC gathered samples collected from sampling sites and hence sorted them to MLC of Medical Group and other 22 inspection institutions in order to complete testing within 24 hours.

After sampling and testing procedures, there were two main jobs to follow-up. On one hand, ITC upgraded the information system for numerous requests by residents to access testing results on mobile applications; on the other hand, the community health-care centers were responsible for health management for residents who need to be quarantined and isolated to observe their physical and mental situation.

## Results

For the screening results, the screening program was completed in 10 days from August 14 to August 23, 2020. In terms of the nucleic acid test, a total of 437,100 samples had been tested and 3 asymptomatic positive cases were identified. Particularly, 2 identified asymptomatic cases were close contacts of the index case, and the third asymptomatic case was the close contact of the first diagnosed asymptomatic patient in this screening program. The residents, who got positive results in either the nucleic acid testing or serological antibody testing, and their close contacts were required to isolate or quarantine for at least 14 days until they got negative results in nucleic acid testing.

In turn of the organization ability of the Hospital Group, 92 sample collection sites were functioning and 4715 person-times of medical staff were involving in total from the integrated healthcare system during these ten days. The maximum number of sample collection site functioning in a single day was 62. Besides, the inspection ability of nucleic acid testing had reached to 27,937 specimens per day.

In addition, the total cost of this regional screening for the sporadic situation was 33,593,490 CNY and the screening cost per capita was 76.86 CNY. The majority of the cost was inspection costs (49.8%), followed by labor costs (29.1%) and material costs (19.0%) (see ***Table 2***). To breakdown the inspection costs (see ***Table 3***), there were 163,322 specimens (accounting for 37.4% of the total) tested in the Hospital Group’s MLC, while 273,778 specimens sent out for inspection (accounting for 62.6%). The testing cost of Hospital Group’s self-inspection and outsourcing inspection was 22.82 CNY and 47.50 CNY per capita respectively, which computed that the outsourcing inspection cost was 2.08 times of self-inspection. There was no significant difference in demographic characteristics (age, gender ratios, and native place distribution) between the two groups (p > 0.05).

**Table 2:** The cost breakdown of the large-scale screening program.


ITEMS	AMOUNT (THOUSAND CNY)	PROPORTION AMONG TOTAL COSTS (%)

**Labor cost**	**9,766.20**	**29.1**

Personnel cost (Daily salary)	5,266.20	53.9

Temporary wage subsidy	4,500.00	46.1

**Material cost**	**6,367.67**	**19.0**

Medical protective supplies	3,427.10	53.8

Sampling supplies	2,750.00	43.2

Logistics supplies	190.57	3.0

**Inspection cost**	**16,732.10**	**49.8**

Hospital Group’s self-inspection cost ^a^	3,727.10	22.3

Outsourcing inspection cost ^b^	13,005.00	77.7

**Informationization cost**	**551.52**	**1.6**

Information equipment ^c^	422.87	76.7

Wireless network card ^d^	127.48	23.1

Software developments ^e^	1.18	0.2

**Other costs**	**176.00**	**0.5**

Transportation fee	40.00	22.7

Accommodation fee	136.00	77.3

**Total**	**33,593.49**	**100**


*Note*: ^a^ The composition of self-inspection cost comprised labor cost in inspection department, equipment depreciation fee for inspection, and the relevant inspection materials costs. The self-inspection in this study was not in line with the charge standard in hospitals, which was calculated according to the actual costs that happened instead of the charging fee set by the local government in hospitals. ^b^ The outsourcing inspection cost was calculated from the unit price and discount provided by the third inspection parties. The final cost is based on the actual number of outsourcing specimens. ^c^ The information equipment includes laptop, scanner, barcode printer, server and storage. The period of depreciation of laptops and PDA equipment was 6 years, and the cost was included according to the actual working hours (10 days) in the cost calculation, namely, 31.88 CNY/10 days for each laptop, and 925.93 CNY/10 days for each server and storage. ^d^ The wireless network card is disposable consumables. ^e^ It was expected that the available period of this software system was 6 years, and the cost was included according to the actual working hours (10 days) in the cost calculation. In addition, since the system was developed by the Hospital Group, the maintenance work was undertaken by the staff within the Hospital Group and there was no breakdown of maintenance cost, so the maintenance cost was not included in this study.

**Table 3:** Screening program inspection cost breakdown and demographic characteristics of inspected populations.


INSPECTION APPROACH	NUMBER OF SPECIMENS	INSPECTION COST (THOUSAND CNY)	INSPECTION COST PER CAPITA (CNY)	PROPORTION IN THE TOTAL NUMBER OF SPECIMENS	PROPORTION OF TOTAL INSPECTION COST

**Hospital Group’s self-inspection**	163,322	3,727.1	22.82	37.4%	22.3%

	Variables		Descriptive statistics		

	Age (year old)		34.44 ± 14.07		

	Gender	Male		50.33%	

		Female		49.67%	

	Native place distribution (inside vs. outside)	Luohu District		3.46%: 96.54%	

		Shenzhen City		10.82%: 89.18%	

		Guangdong Province		49.95%: 50.05%	

**Outsourcing inspection**	273,778	13,005.0	47.50	62.6%	77.7%

	Variables		Descriptive statistics		

	Age (year old)		32.89 ± 15.23		

	Gender	Male		51.02%	

		Female		49.98%	

	Native place distribution (inside vs. outside)	Luohu District		3.76%: 96.24%	

		Shenzhen City		11.58%: 89.42%	

		Guangdong Province		55.00%: 45.00%	

**Total**	**437,100**	**1,673.21**	**38.28**	**100.0%**	**100.0%**


## Discussion: results analysis and experience learned

The large-scale screening program was completed in 10 days and screened nearly 437,100 samples. The Hospital Group completed 163,322 self-inspection samples, achieving an average daily inspection ability of 16,000 samples in August 2020. After this screening program, except for 3 asymptomatic cases were screened, no more positive results were presented in the following medical observation period. Some might doubt the existence of false-negative results in this program due to the low level of virus in asymptomatic infected individuals, nonstandard operation in collection or inspection process, and low accuracy of testing assay approach [20]. However, the official reports showed that all the diagnosed infected cases happened in Shenzhen after this screening (including 19 new symptomatic cases and 149 new asymptomatic cases until January 8, 2021) were imported from abroad, which demonstrates the effectiveness of the large-scale screening program in blocking the route of infection among communities.

### For the integrated care system: the capabilities increased in coping with public health problems by integrating regional medical resources

**Figure 3:**
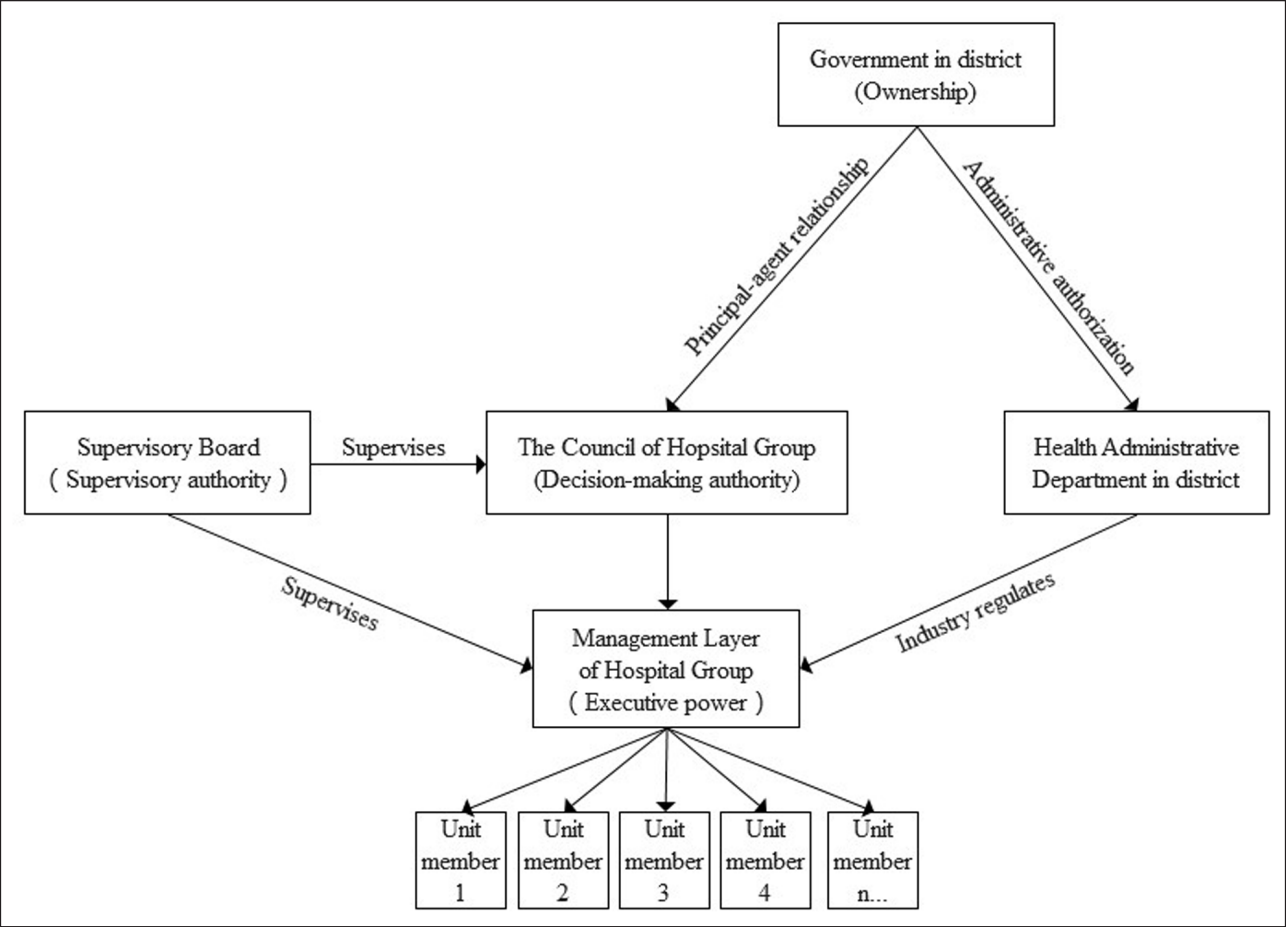
The internal governance structure of Hospital Group and its relationships with governments and relevant departments.

Concerning the timeliness and accuracy requirements of a large-scale screening program, it might be advantageous to conducted by the Hospital Group compared with the traditional medical institution independent of other institutions. The public hospitals and community healthcare centers in Luohu district were operated independently until 2015 when the integration started and the Hospital Group was established. Two main efforts made the strong relationship between medical institutions in Hospital Group at the beginning. The first effort is administrative management reform of managing all medical institutions in Luohu district by the only one legal representative and improving the internal governance structure (see ***Figure 3***). Firstly, the Council of Hospital Group was established and built a principal-agent relationship with the government in district. The government owns the ownership of public hospitals and public community healthcare centers, entrusts the Council of Hospital Group to perform the decision-making power to manage the Hospital Group. the Council has the highest decision-making authority and nominates the presidents of the Hospital Group. Secondly, the Management layer owns the executive power, in which the president of Hospital Group is the only legal representative of health institutions in the integrated healthcare system. The president of the Hospital Group is responsible for the specific operation and management of the medical institutions of Hospital Group within the scope authorized by the Council and thus the Hospital Group has a high degree of independent operation autonomy. Thirdly, a board of supervisory was established to supervise the Council and the management layers of the Hospital Group. In the meanwhile, the district health administrative department regulates Hospital Group in industry standards to ensure its commonweal as a non-profit organization.

The second effort is a payment reform of “global budget and balance retention” in Hospital Group. “Global budget” means that the new medical insurance payment mechanism packages the annual budget of medical insurance fee for Hospital Group according to the total amount of inpatient medical insurance of contracted residents plus the annual municipal average growth rate happened in last year; “Balance retention” means that the year-end settlement savings of packaged budget are paid to Hospital Group, which indicates that if the contracted residents spend less money on medical treatments, Hospital Group can receive more profits. Also, contracted residents can freely choose a medical institution and the medical insurance expenditure incurred in other hospitals (i.e. medical institutions outside the Hospital Group) will be paid from the packaged budget. This regulation forces Hospital Group to develop self-capability in medical and health services to increase population voluntarily of seeking help and hospitalizing in Hospital Group.

Thus, the Hospital Group builds a firm collaborative relationship between 5 public hospitals and 37 community healthcare centers through the benefit-sharing and one legal representative mechanism. Furthermore, the Hospital Group deepens the integration of resources to optimize the allocation of medical resources among various hospitals in the jurisdiction. One of the typical and successful integration outcomes is the MLC, which played a crucial role in the large-scale screening program, testing 37.4% of collected specimens in this screening program. The daily nucleic acid testing ability was up to 27,937 specimens in August 2020, whose testing ability in nucleic acid testing ability has increased by two times than before the reform. The inspection ability is uneven in China at the corresponding period, for example, the maximum testing number in Heilongjiang, Shandong, Ningxia, and Shaanxi Province is 20 000, 10 000, 16 000, and 50 000 per day respectively [21,22,23,24]. When compared with the foreign country, a mass screening conducted in Luxembourg with the peaking testing amount was 20,000 tests per day [15]. The inspection ability in our integrated healthcare system is relatively advantaged by comparison, which could be used to improve other clinical and technical aspects in the Hospital Group. Therefore, the effectiveness of integrating regional medical resources in developing medical capability should be emphasized.

### For the large-scale screening program: measures to lower the screening cost

As for the cost of screening, this study provided a reference for other cities in China or worldwide that need to conduct a large-scale screening or compare the screening cost in different working strategies. However, it is too early to discuss the cost-effectiveness of large-scale screening before the spread of epidemic being controlled, which means that considering approaches to minimize the cost of large-scale or mass screening would be more valuable at this stage. Thus, this study summarizes some suggestions that might lower the screening cost from inspection, information construction, and labour aspects. For example, the pooled sampling could reduce the inspection cost when applying in community screening. At the end of 2020, up to 8 in 1 pooled sampling testing technology have been clinically verified and promoted their applications in COVID-19 prevention and control [25]. In this screening program, a total of 2,478 five-in-one pooled sampling tubes were detected and a total of 18,025 person-times were tested, which had no false-negative results. Therefore, in the large-scale screening program, we can reduce per capita costs of consumables, inspection, and time through conducting the mixed sampling test in low-risk populations, which can also rapidly screen infected patients and quickly control the outbreak.

Subsequently, the Hospital Group collected environmental samples in this screening program to decide whether the infected source of index case came from food or environmental transmission; similarly, the surveillance of environmental sample (e.g. waste-water of communities) can be an indicator for implementing testing in individual or community adopted in both emergent screening or mass screening [26]. A previous report about COVID-19 from a college in USA showed that a positive water sample from a dorm in college might contribute to averting an outbreak in college by diagnosing 2 asymptomatic students in this dorm. Therefore, the novel testing techniques and increased testing capability are strongly support the large-scale screening in sporadic epidemics and mass screening in normal prevention job.

### For the COVID-19 pandemic control: asymptomatic cases and their close contacts should be quarantined strictly

The necessity of large-scale screening is a controversial issue because of the uncertain transmission ability of asymptomatic patients in communities. Some experts suggested that the transmission risk of asymptomatic cases is weaker than symptomatic patients because of the lower quantity of viral loads and shorter duration of the infective period [27]. Another study illustrated that the perniciousness of the virus might decrease over time and thus more infected people become asymptomatic instead of symptomatic [28]. However, a close contact of 2 diagnosed asymptomatic patients was identified as an asymptomatic case in this screening program, which might support that the asymptomatic patients are infectious, and most newly infected people tend to be asymptomatic. This finding was supported by another study in Nanjing of which reported one of the asymptomatic patients infected his cohabiting family members [13]. In addition, a mass screening in Luxembourg showed that the asymptomatic and symptomatic carriers infected average the same number of people [15]. Thereby, the screening strategies should be stressed for both symptomatic and asymptomatic carriers, and the lager-scale screening is useful to comprehensively screen infected people out.

## Conclusion

Large-scale screening is a significant measure to detect nascent infection chains and prevent outbreaks. As the asymptomatic carriers are found to be infectious, their close contacts need to be quarantined and screened carefully. Regular surveillance of environmental sampling and fever clinics is essential for epidemic prevention and control in the post-pandemic period; while rapid response capability, sufficiently available medical resources, and standardized screening procedures are decisive for large-scale screening in the sporadic epidemic.

Achieving effective screening in emergency response programs requires not only efficient and accurate sampling and testing but also the adequate coverage of screening objectives, which could be satisfied by the integrated care system.

On one hand, as the integrated care system has gathered regional medical resources from 5 public hospitals, a personnel list against the outbreak of COVID-19 with well-educated about sampling and testing techniques could be arranged before the sporadic epidemic. It also can deploy regional medical staff and material as needed to overcome the sporadic epidemic of infectious disease without affecting the accessibility of normal medical services for regional residents. Additionally, the medical capability of the Hospital Group has greatly developed through integration that can effectively support the large-scale screening program. For example, the MLC has been established once the integrated care system gathered inspection resource and awarded the ISO15189 international standard laboratory certificate in 2019, which not only is responsible for all the medical testing program in the Hospital Group but also serves as a third-party medical laboratory providing outsourcing medical testing services to other health institutions. Besides, the Hospital Group invested 4715 person-time in this screening program, including 3607 front-line sampling and testing personnel and 1298 support personnel, which could not be achieved without personnel integration and could not be arranged orderly without the HRC. Thus, with the available medical sources and enhanced medical capability, the emergent screening program can complete efficiently.

On the other hand, the integrated care system involves both public hospitals and community healthcare centers in the district, with the priority of developing community healthcare centers to strengthen the primary care and homogenize the medical services capability in the district. As believe that community engagement is crucial for COVID-19 response strategy [29], the Hospital Group comprises more than 30 community healthcare centers that show great advantages in this screening program. Firstly, the medical staff in community healthcare centers participated in the sampling and contact tracing as the contextual factor and did complement the shortage of public hospitals and CDC in preparedness and follow-up procedure of screening program. Secondly, the Hospital Group sets up numerous Family Doctor groups (similar to the GP in England) based on community healthcare centers to in charge of the health management for residents. Since the medical capability and affordability of community healthcare centers have been greatly improved, not only the residents are more willing to obey GPs’ suggestions to adopt sampling and required quarantine, but also the medical staff in community healthcare centers can support the screening program as surveillance to complete successfully.

In conclusion, the integrated care system enhances medical capability and increases working efficiency as well as develops the regional primary care ability to support effective screening. However, this study exists several limitations. For example, since the screening program was triggered by diagnosing a symptomatic case, the screening program mainly detected the residents who might exposure to the infected danger living around and thus the results could only contribute to the incident rate of COVID-19 in Luohu district rather than in Shenzhen City.

## Additional File

The additional file for this article can be found as follows:

10.5334/ijic.5941.s1 Supplementary Text 1. Details of the Screening Procedure.
