# Supplementary material for: A Large-Scale Screening Responding Sporadic Epidemic of COVID-19 in China by an Integrated Health-Care System
Source: Int J Integr Care. 2022 Mar 23;22(1):23. doi: 10.5334/ijic.5941 (PMC8954885; doi:10.5334/ijic.5941)
Supplement: Supplementary Text 1. — Details of the Screening Procedure. [file ijic-22-1-5941-s1.pdf]

**Supplementary Material to manuscript 'A Large-scale Screening Responding Sporadic Epidemic of COVID-19 in China by an Integrated Health-care System'**

The supplementary material contains one text content to complement the details about the procedure of screening programme.

**Supplementary Text 1.** Details of the Screening Procedure

1 Preparedness stage

1.1 Reservation and accumulation for sufficient capacity

The symptomatic and asymptomatic imported cases appear constantly in Shenzhen due to the special geographic and economic characteristics of Shenzhen. the Hospital Group appeals to the medical institutions in integrated health-care system reserved sufficient capacity to cope with the epidemic prevention.

The resource-sharing centers and administrative centers, especially the Human Resources Center (HRC) and the Medical Laboratory Center (MLC), are closely associated with the effective control of COVID-19 pandemic.

The Group's HRC, who in charge of the human resource management to coordinate the personnel among all the medical institutions within the integrated health-care system, developed a list and a formal document to guarantee that the district has sufficient manpower to address the sudden epidemic. This structure is an important institutional basis for the rapid, flexible allocation of human resources for public health emergencies. Alongside, the Group's MLC was established in 2015 by merging the inspection resources of various medical institutions in Luohu District, which has awarded ISO15189 international standard laboratory certificate. The MLC offers a 24/7 emergency response testing service for cross-border drivers from Hong Kong during the pandemic period, which enables MLC to accumulate inspection experience in nucleic acid testing and response timely in emergent outbreaks.

1.2 Exploration of the informational platform for epidemic prevention and control

The hospital group plays a major role in nucleic acid testing for cross-border drivers or residents at 4 main ports in Shenzhen after the outbreak of COVID-19 at the beginning of 2020. To facilitate the access to epidemiological information of cross-border personnel, the group established an interface between the hospital information system (HIS) and the

e-government system, and to get through the information acquisition channels among multiple departments. Moreover, with this interface, the results of nucleic acid testing at ports can update to the e-government system, which contributes to real-time data sharing and collaboration.

## 2 Screening stage

### 2.1 Establishment of the leading team

After receiving the formal information about the diagnosed symptomatic case in a supermarket, a specialized task team for leading the screening programme was formed immediately, who was responsible for formulating screening strategies, communicating with relevant departments, and supervising the whole process.

### 2.2 Deployment of the human and materials resources

One of the advantages of integrated healthcare system is deploying human and materials resources to organize the assigned sample collection teams. To ensure sufficient personnel, some clinical departments of the Hospital Group (e.g. the stomatological center, Ophthalmology and Otolaryngology department of the general hospital) stopped for 2 days to support the epidemic screening programme. Besides, the logistics distribution center implemented the regulation mode of "unified storage, regional delivery, and on-demand distribution", which means that the center could uniformly dispatch the resources of all the distributors and suppliers contracting with the hospital group. Therefore, in the urgent screening programme, the human resources center and logistics distribution center could allocate the personnel and materials according to the actual situation of each sample collection sites.

During these ten days, 92 sample collection sites were functioning and 4715 person-times of medical staff were involving in total from the integrated healthcare system. The maximum number of sample collection sites functioning in a single day was 62, and each site consisted of a head nurse or GP as the "site leader" who has rich experience in nucleic acid sampling and outstanding management ability, a hospital leader, 2-3 community staff, a personal information collector and 5-6 sampling persons. Despite the designated collecting site, 2 mobile sample collection teams were available for scattered residential buildings or communities with small populations.

## 2.3 Sample collection and transport

To prevent the further spread of the COVID-19, on the one hand, the screening work must be tested as much as possible to ensure coverage and timeliness; on the other hand, it has higher requirements on the quality of sample collection and transport to guarantee the effectiveness of screening.

About the sample collection, most of the sampling staff were temporarily deployed in this emergent action, and hence the standard training for sample person was taken seriously. For example, it is necessary to stay at the sampling place for more than 5 seconds to complete the collection of nasopharyngeal swab and pharyngeal swab sample. Both single sampling and pooled sampling were adopted, but the later one was suitable for residents in low-risk communities.

We stored the samples in temporary storage boxes, which would be transferred by refrigerated delivery vehicle to guarantee the safety and stability of samples, and the transporters should be responsible for checking the number of specimens.

## 2.4 Sample sorting and testing

To complete the inspection work within 24 hours after the sample collection, the MLC in our Group contributed 37.4% of the testing workload while the remaining 62.6% of samples were outsourced to 22 laboratories of municipal medical institutions or third-party inspection institutions. Before distributing samples to inspection laboratories by refrigerated trucks, the logistics distribution center gathered sample collected and hence sorted according to the distance and testing ability of inspection institutions. The advantage of centralized sorting is conducive to the later tracing of positive results and increases the efficiency of population screening by avoiding the separation of personnel information and sample.

## 3 Follow-up stage

## 3.1 Result query and feedback

Within 24 hours after sampling, the testing results would be released and participants could enquire about it in various ways. Residents who were scanned their e-code connected to their ID information on Wechat or Alipay application at the sampling stage can get results on

mobile applications, and those who only provide personal information and mobile number without using e-code would receive a result message; otherwise, participants could only know the testing result from the paper version of nucleic acid test certificate issued by community health-care center using their ID card.

In the result page on mobile applications, apart from the description about the testing result, there would be a colored unique SQ code, in which the green color represents the negative result while the red e-code was a positive result. As of August 24, a total of 295 thousand messages and 13 thousand paper copies had been issued.

## 3.2 Quarantine and surveillance

After sample collection and inspection, the isolation requirement was compulsory for high-risk and sub-high-risk residents. In the duration, the medical staff of community health-care centers in the Group provided daily health service and monitored their physical condition. If the isolated people reported some psychological issue, the psychologist or GP in community health-care centers would undertake psychological interventions for them to get through.

Besides, since the infected source of the first domestic case could not be identified in a short time, there might be other existing people affected by the same source without involving in this screening programme. Consequently, the community health-care center and the fever clinic in hospitals in the Group emphasized the management of fever patients. The fever clinic is a special clinic for preventing and controlling acute infectious diseases setting up by the outpatient department in hospitals during the pandemic period, which is specially applied for screening suspected infectious patients and treating fever patients. During the screening programme, the fever clinic in the Group played the role of the sentinel surveillance and tested all patients having a fever. A total of 1746 fever patients undergoing the nucleic acid test and all testing results were negative.
